# Immunological significance of prognostic alternative splicing signature in hepatocellular carcinoma

**DOI:** 10.1186/s12935-021-01894-z

**Published:** 2021-04-01

**Authors:** Qianhui Xu, Hao Xu, Rongshan Deng, Nanjun Li, Ruiqi Mu, Zhixuan Qi, Yunuo Shen, Zijie Wang, Jingchao Wen, Jiaxin Zhao, Di Weng, Wen Huang

**Affiliations:** 1 The Second Affiliated Hospital and Yuying Children’s Hospital of Wenzhou Medical University, No 109. Xueyuan West Road, Wenzhou, 325000 Zhejiang China; 2 Zhejiang University School of Medicine, Hangzhou, 310009 Zhejiang China

**Keywords:** Hepatocellular carcinoma, Alternative splicing, Tumor immune microenvironment, Prognosis, Immunotherapy

## Abstract

**Background:**

Hepatocellular carcinoma (HCC) ranks the sixth prevalent tumors with high mortality globally. Alternative splicing (AS) drives protein diversity, the imbalance of which might act an important factor in tumorigenesis. This study aimed to construct of AS-based prognostic signature and elucidate the role in tumor immune microenvironment (TIME) and immunotherapy in HCC.

**Methods:**

Univariate Cox regression analysis was performed to determine the prognosis-related AS events and gene set enrichment analysis (GSEA) was employed for functional annotation, followed by the development of prognostic signatures using univariate Cox, LASSO and multivariate Cox regression. K-M survival analysis, proportional hazards model, and ROC curves were conducted to validate prognostic value. ESTIMATE R package, ssGSEA algorithm and CIBERSORT method and TIMER database exploration were performed to uncover the context of TIME in HCC. Quantitative real-time polymerase chain reaction was implemented to detect ZDHHC16 mRNA expression. Cytoscape software 3.8.0 were employed to visualize AS-splicing factors (SFs) regulatory networks.

**Results:**

A total of 3294 AS events associated with survival of HCC patients were screened. Based on splicing subtypes, eight AS prognostic signature with robust prognostic predictive accuracy were constructed. Furthermore, quantitative prognostic nomogram was developed and exhibited robust validity in prognostic prediction. Besides, the consolidated signature was significantly correlated with TIME diversity and ICB-related genes. ZDHHC16 presented promising prospect as prognostic factor in HCC. Finally, the splicing regulatory network uncovered the potential functions of splicing factors (SFs).

**Conclusion:**

Herein, exploration of AS patterns may provide novel and robust indicators (i.e., risk signature, prognostic nomogram, etc.,) for prognostic prediction of HCC. The AS-SF networks could open up new approach for investigation of potential regulatory mechanisms. And pivotal players of AS events in context of TIME and immunotherapy efficiency were revealed, contributing to clinical decision-making and personalized prognosis monitoring of HCC.

**Supplementary Information:**

The online version contains supplementary material available at 10.1186/s12935-021-01894-z.

## Introduction

Hepatocellular carcinoma (HCC) is a malignant and aggressive disease marked with frequently diagnosed and high cancer-attributable mortality in the world [1–3]. Despite great advance in HCC early diagnosis and anti-tumor therapy, clinical treatment result is still undesirable [4–6]. Both tumor, node, metastasis (TNM) categories and Barcelona Clinic Liver Cancer (BCLC) staging classification, which were widely used prognostic tools, failed to precisely predict results of patients with same clinicopathological stage [7–9]. Furthermore, the high heterogeneity of HCC remains great challenge against therapeutic benefits, which makes it difficult to accurately predict clinical result [1, 10, 11].

A great part of HCC was derived from inflammatory liver diseases, which suggested that infiltrating immune cells in tumor immune microenvironment (TIME) might serve as pivotal regulatory roles in tumorigenesis and progression in HCC [12–14]. Recently, immunotherapy has received extensive attention since it yielded encouraging results in multiple malignancies [15]. However, only 20% of HCC patients were observed objective response to immunotherapy according to preclinical trials [16]. Hence, the most effective strategy for precise prognostic predictions of how a given malignancy will respond to immunotherapy or tumor will progress may be one based on molecular risk distribution, identifying HCC patients on line with particular molecular signatures, enhancing prognostic precision and optimize immunotherapeutic benefit accordingly.

Alternative splicing (AS) defined as the mechanism by which edit pre-mRNA to produce mature mRNA, greatly contributed to the complexity of genome and the diversity of proteome [17, 18]. It is well established that AS events included such main patterns as alternate acceptor site (AA), alternate donor site (AD), alternate promoter (AP), alternate terminator (AT), exon skip (ES), retained intron (RI) and mutually exclusive exons (ME) [19]. Unbalanced expression of AS occurs frequently in cancer and received extensive attention as pivotal role in tumor initiation, development, metastasis and response to treatment [20–22]. Besides, splicing factors were found to act as a vital player in the regulation of AS events [23]. It was worth mentioned that aberrant expression of crucial splicing factors can lead to the oncogenic splicing isoforms [24, 25].To date, multiple researches had been performed to unravel the biological relevance and clinical significance of AS events in HCC [26–28].

There have been several articles focusing on AS-based prognostic model [29–31], however, the correlation of AS prognostic signature with TIME and immunotherapy remains obscure.

Thus, it is imperative to perform a comprehensive analysis of AS events to uncover the characterization of TIME and underlying molecular mechanisms of tumorigenesis, further optimize clinical benefits.

In this study, the AS pattern of TCGA LIHC cohort was outlined and AS events associated with the survival was determined through comprehensive bioinformatic analysis. Next, AS-based prognostic signatures were developed then validated, and an AS-clinicopathologic nomogram was constructed to facilitate clinical application. Then, the correlation of prognostic signature with the complexity of TIME and immunotherapeutic efficacy was investigated. Additionally, the potential role of ZDHHC16 in HCC was explored. Finally, an AS-SFs regulatory network was established to elucidate the potential mechanism involving in HCC progression.

## Materials and methods

### Multiomic data acquisition

The transcriptome information and survival information of the HCC patients were downloaded from The Cancer Genome Atlas (TCGA) portal (http://cancergenome.nih.gov) for subsequent analysis. The alternative splicing data of TCGA LIHC-cohort were obtained from SpliceSeq (http://bioinformatics.mdanderson.org/TCGASpliceSeq). Samples were screened when setting PSI value exceeds 0.75 as filter cut-off point. All analyses were performed based on the publication guidelines of TCGA. The analysis process flow chart was presented in Additonal file 1: Figure S1.

### Process of AS profile identification

In TCGA splice-seq, the percent spliced in (PSI) value to quantify AS events were detected then calculated. By using UpsetR package, Upset plot was delineated to discovery the seven subtypes of AS events (alternate acceptor site (AA), alternate donor site (AD), alternate promoter (AP), alternate terminator (AT), exon skip (ES), mutually exclusive exons (ME), and retained intron (RI)). The AS events were annotated by combining the splicing type, ID number in the SpliceSeq and the corresponding parent gene symbol. For example, in “MRPL43|12,849|AT”, MRPL43 denotes the corresponding parent gene name, 12,849 represents the ID of splicing variant and AT indicates the splicing type.

### Identification of survival-related AS events

When the standard deviation of PSI value is less than 0.01, the AS data were excluded. Univariate Cox regression analysis was carried out to detect the association between AS events and overall survival (OS) of HCC patients (Additonal file 2: Table S1), which were presented with the UpSet plot and the volcano map. Besides, the top 20 most significant AS events from the seven subtypes were summarized in the bubble charts.

### Construction and validation of prognostic signature

Firstly, Lasso regression analysis was employed to determine candidates in each splicing pattern and avoid model over-fitting. Secondly, the identified AS events were introduced into Multivariate Cox regression analysis to screen the prognostic predictor. Given the mode of AS events is independent from each subtype in post-transcriptional modification, the identified AS events in each splicing subtype above were integrated to generate another prognostic signature. Subsequently, the risk scores were calculated according to each prognostic predictor and the formula for computing the risk score is as follows: Risk score = βAS event1 × PSIAS event1 + βAS event2 × PSIAS event2 + ⋯ + βAS eventn × PSIAS eventn. The specific formulas of each signature were presented in Additonal file 2: Table S2. Based on the median value of risk score, patients were ranked into low-risk group and high-risk group. Kaplan–Meier survival curves were analyzed with “survival” R package. Then, the time-dependent receiver operating characteristic (ROC) curves were performed to examine the prognostic value of this signature. Besides, univariate and multivariate Cox regression were analyzed to confirm whether the signature can serve as an independent factor for prognostic prediction. Additionally, stratified survival analysis was conducted to further validate the prognostic performance independent from such clinical characteristics as age, gender, tumor stage, pathological grade, T category, N category and M category.

### Construction of prognostic nomogram

To comprehensively assess prognosis predictive ability of risk signature, tumor stage, gender, age, WHO grade, T category, N category and M category for 1/2/3-year OS, time-dependent receiver operating characteristic (ROC) curves was perform to calculate the area under the curve (AUC) values [32]. To contribute a quantitative manner to predicting overall survival of patients with HCC, prognostic nomogram which containing AS-based risk model and other clinical variables was established to estimate 1‐, 2‐and 3‐year overall survival probability. Subsequently, the calibration curve which shown the prognostic value of as-constructed nomogram was delineated. A calibration curve close to 45° is an indication of good prediction ability of the model constructed by this factor.

### Correlation of risk score with tumor infiltrating immune cells characterization

Immune infiltration information consists of every specimen immune cell fraction (i.e., B cells, CD4 + T-cells, CD8 + T-cells, dendritic cells, macrophages, and neutrophils, etc.,) were downloaded from tumor immune estimation resource (TIMER) (https://cistrome.shinyapps.io/timer/). The correlation between tumor immune cell infiltrating with the prognostic risk score was performed. A single sample gene-set enrichment analysis (ssGSEA) was implemeted to elucidate the enrichment of the two distinct risky subgroups in 29 immune function‑associated gene sets via invoking the R package “GSEAbase”. Subsequently, the R package “ESTIMATE” was employed to assess tumor purity and the extent and level of infiltrating cells, namely stromal cell and immune cell, that could validate significant distinct tumor immune microenvironment characterization between two risky subgroups. The fraction of 22 immune cell types for each tumor specimen was developed through cell type identification by estimating relative subsets of RNA transcripts (CIBERSORT; https://cibersort.stanford.edu/).

### Role of risk score in immune checkpoint blockade treatment

Refer to existing studies, expression level of immune checkpoint blockade-related key genes might be correlated with clinical outcome of immune checkpoint inhibitors blockade treatment [33]. Herein, six key genes of immune checkpoint blockade therapy: programmed death ligand 1 (PD‐L1, also known as CD274), programmed death ligand 2 (PD‐L2, also known as PDCD1LG2), programmed death 1 (PD‐1, also known as PDCD1), cytotoxic T‐lymphocyte antigen 4 (CTLA‐4), indoleamine 2,3‐dioxygenase 1 (IDO1), and T‐cell immunoglobulin domain and mucin domain‐containing molecule‐3 (TIM‐3, also known as HAVCR2) in HCC [34–36] were extracted. To elucidate the potential player of as-constructed risk signature in ICB treatment of HCC, AS-based prognostic signature and expression level of six immune checkpoint blockade key genes were correlated. Finally, the expression level of 47 immune checkpoint blockade-related genes (i.e., PDCD1, etc.,) between low-/high-risk groups were compared.

### Construction of splicing regulatory network

A list of 404 splicing factors (SFs, Additonal file 2: Table S3) was referred to a previous research [37] and the RNA-seq profiles of SFs were downloaded from the TCGA database. The Spearman correlation analysis was performed to evaluate the association between the SFs and the survival-relevant AS events (Additonal file 2: Table S4). P < 0.001 and Correlation coefficient > 0.6 was the cutoff values. Finally, Cytoscape (version 3.8.0) was employed to build an underlying SF-AS regulatory network.

### Experimental validation

L02 cell (human hepatic cell line) and two human HCC cell lines (MHCC-97H cells and HCC-LM3) were purchased from the Cell Bank of the Type Culture Collection of the Chinese Academy of Sciences, Shanghai Institute of Biochemistry and Cell Biology. The cell lines were all cultured in Dulbecco’s minimum essential media (DMEM) plus 10% fetal bovine serum (FBS; Invitrogen, Carlsbad, CA, USA). All cell lines were grown without antibiotics in a humidified atmosphere of 5% CO2 and 99% relative humidity at 37℃. Three different cell lines were subjected to quantitative real-time polymerase chain reaction (qRT-PCR).

### RNA isolation and qRT-PCR analysis

Total RNA was extracted from cells using TRIzol (Invitrogen, Carlsbad, CA, USA) according to provided instructions. RNA concentration and purity were measured in triplicates utilizing the NanoDrop 2000 spectrophotometer (Thermo Scientific Inc., Waltham, MA, 93 USA). Then, total RNA was reverse transcribed to cDNA using the cDNA Reverse Transcription Kit (Vazyme, Nanjing, China). To determine the expression of ZDHHC16, cDNAs were subjected to qRT-PCR using SYBR Green Real-time PCR Master Mix (Takara) in Applied Biosystems 7500/7500 Fast Real-Time PCR System (Thermo Fisher Scientific). All samples were analyzed in triplicates. Glyceraldehyde-3-phosphate dehydrogenase (GAPDH) levels were used as the endogenous control and relative expression of ZDHHC16 was calculated using the ${2}^{{ - \Delta \Delta {\text{C}}_{{\text{t}}} }}$ method. The sequences of primers used for PCR were as follows: ZDHHC16, 5′-CCACCAGACTCCACCACCTACC -3′ (forward) and 5′-GCCACAGAACTGCACAGGAACC -3′ (reverse); and GAPDH, 5′-CAGGAGGCATTGCTGATGAT-3′ (forward) and 5′-GAAGGCTGGGGCTCATTT-3′ (reverse).

### Statistical analysis

The Wilcoxon test was employed to compare two groups, whereas the Kruskal–Wallis test was carried out to compare more than two groups. Overall survival (OS) refers to the interval from the date of diagnosis to the date of death. Survival curves were plotted via the Kaplan–Meier log rank test. Risk scores, clinical variables, immune cell infiltrating extent and immune checkpoints were correlated with Pearson correlation test. CIBERSORT algorithm results with *p* >  = 0.05 were rejected for further analysis. Univariate and multivariate analyses were performed via Cox regression models to validate the independent prognosis predictive performance of risk signature. The prognostic value of the AS-based signatures for 1-, 2- and 3-year OS was assessed with the ROC curves. *p* < 0.05 deemed as statistical significance. R software (version 4.0.2) was utilized for all statistical analyses.

## Results

### Clinical characteristics and integrated AS events profiles in HCC

377 HCC patients were obtained using the TCGA database, and seven patients with incomplete information were excluded from this study. In total, 370 patients were enrolled. The basic clinical information of patients is shown in Table 1. The AS events profiles were comprehensively analyzed and the gene intersections among the seven subtypes of AS events was presented in UpSet plot (Additonal file 1: Figure S2A). These results showed that ES was the predominant splicing pattern meanwhile AD marked as the least frequent.

**Table 1:** Baseline data of all HCC patients

Characteristic	Type	*n*	Proportion (%)
Age	< = 65	235	62.33
	> 65	141	37.40
	unknow	1	0.27
Gender	FEMALE	122	32.36
	MALE	255	67.64
Grade	G1–2	235	62.33
	G3–4	137	36.34
	unknow	5	1.33
Stage	Stage I–II	262	69.50
	Stage III–IV	91	24.14
	unknow	24	6.37
T stage	T1–2	280	74.27
	T3–4	94	24.93
	unknow	3	0.80
M stage	M0	272	72.15
	M1	4	1.06
	unknow	101	26.79
N stage	N0	257	68.17
	N1	4	1.06
	unknow	116	30.77

### Identification of the survival-relevant AS events

**Fig. 1:**
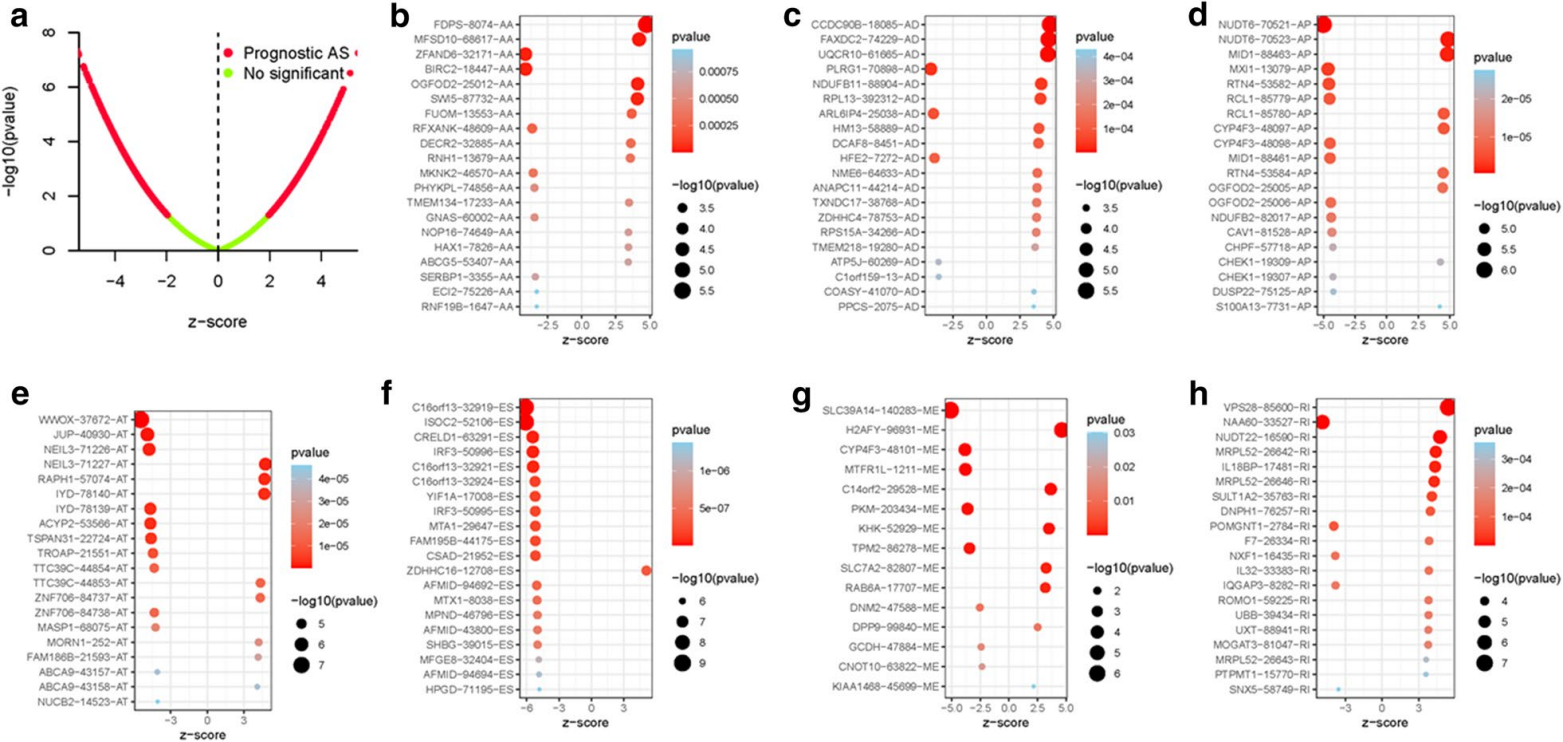
The survival-relevant AS events. **a**) The volcano plots of survival-relevant AS events. The most significant survival-relevant AAs, ADs, APs, ATs, ESs, MEs and RIs in TCGA LIHC cohort (**b**–**h**)

**Fig. 2:**
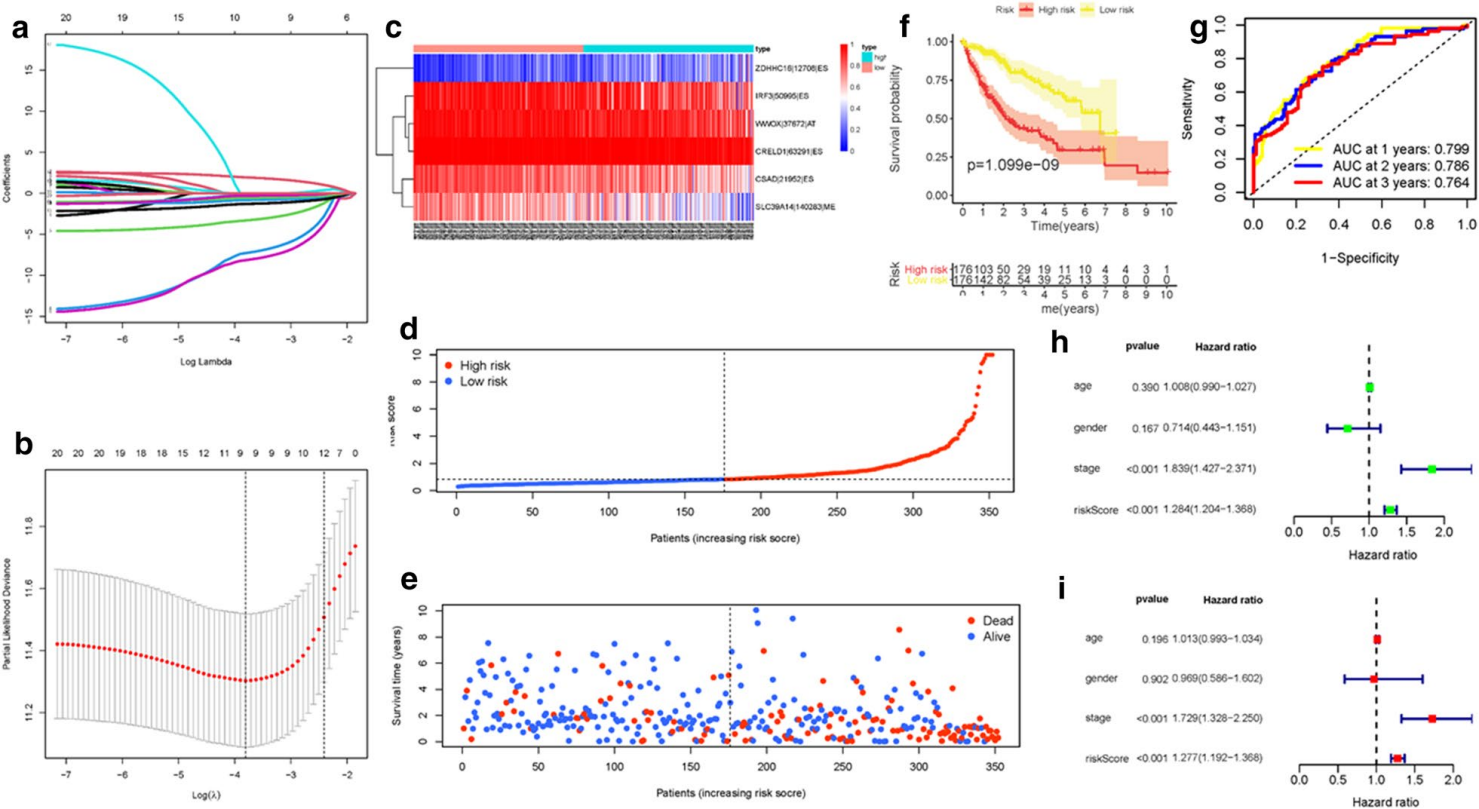
Confirmation of ALL AS-based prognostic signature. **a** LASSO coefficient profiles of the whole AS events. **b** Ten‐time cross‐validation for tuning parameter selection in the lasso regression. **c** Heatmap of the ALL signature AS events PSI value in HCC. The color from red to blue shows a trend from high expression to low expression. **d** Distribution of ALL signature risk score. **e** The survival status and duration of HCC patients. **f** Kaplan–Meier curve presenting survival in the high-risk and low-risk sets. **g** ROC analysis of the risk scores for overall survival prediction. The AUC was calculated for ROC curves, and sensitivity and specificity were calculated to assess score performance. Proportional hazards model results. **h** Univariate Cox regression results. **i** Multivariate Cox regression results

With the help of Univariate Cox regression analysis, a total of 3294 AS events which were significantly related with survival were identified (*p* < 0.05). The detailed description was recorded in TAdditonal file 2: able S1. The intersecting sets of genes and splicing subtypes were delineated in the UpSet plot (Additonal file 1: Figure S2B). Among these subtypes of AS events, ES was the predominant pattern. The volcano map was generated to display the AS events (Fig. 1a). The first 20 significant survival-related AS events from the seven subtypes were summarized in Figs. 1b–h, 2.

### Development of the prognostic signature

Stepwise Lasso algorithm and multivariate Cox regression analysis were employed to estimate the prognostic performance of these identified survival relevant AS events. The results of Lasso regression analysis including seven subtypes of AS events and amalgamated AS events with seven splicing types were displayed in Additonal file 1: Figures S3A–S3G, S4A–S4G and 2A–2B. Then, multivariate Cox analysis was implemented to determine optimal survival-relevant AS events. Lastly, eight AS (AA, AD, AP, AT, ES, ME, RI, and ALL) prognostic signature were constructed. Table 2 presented the detailed formulas of each signature.

### Confirmation of the prognostic signature

Based on the cut-off value of median risk score, HCC patients were stratified into low and high-risk subgroups for further research. Heatmaps displayed the distributions of AS events PSI values with corresponding subgroups and patients (Additonal file 1: Figures S5A, S5D, S6A, S6D, S7A, S7D, S8A and 2C). The allocations of risk score (Additonal file 1: Figures S5B, S5E, S6B, S6E, S7B, S7E, S8B and 2D) and dot pot of survival status (Additonal file 1: Figures S5C, S5F, S6C, S6F, S7C, S7F, S8C and 2E) suggested that high-risk HCC patients had shorter overall survival. Besides, Kaplan–Meier curve corroborated that patients with low-risk possessed significant better prognosis than patients in high-risk group (Additonal file 1: Figures S9A, S9C, S9E, S9G, S10A, S10C, S10E and 2F; all P < 0.05). To assess the prognostic value of risk signatures in HCC cohort, ROC curve were further analyzed. Area under curves of risk scores at 1-, 2- and 3‐year survival times were all more than 0.75, suggesting great sensitivity and specificity of their survival predictive ability (Additonal file 1: Figures S9B, S9D, S9F, S9H, S10B, S10D, S10F and 2G). Besides, results of univariate Cox model (Additonal file 1: Figures S11A, S11B, S11C, S11D, S11I, S11J, S11K and 2H) and multivariate Cox regression analysis (Additonal file 1: Figures S11E, S11F, S11G, S11H, S11L, S11M, S11N and 2I), suggesting risk scores could act an independent indicator in HCC.

A stratification analysis was employed to validate whether ALL prognostic signature still had powerful prognostic predictive ability when HCC patients classified into various subgroups based on clinical characteristics. Relative to patients with low-risk, high-risk HCC patients presented poorer prognosis in both the early- and late-stage subgroups (Additonal file 1: Figures S12A, S12B). Similarly, ALL prognostic signature presented excellent prognostic prediction performance for patients in T1-2 or T3-4 status (Additonal file 1: Figures S12C and S12D), patients male or female gendered (Additonal file 1: Figures S12E, S12F), patients in 1–2 or 3–4 tumor grade (Additonal file 1: Figures S12G, S12H), patients aged <  = 65 years or > 65 years (FigAdditonal file 1: ures S12I, S12J), patients in N0 status (Additonal file 1: Figures S12K), and patients in M0 status (Additonal file 1: Figures S12L). These results suggested that it can be an outstanding predictor independent from clinical parameters in patients with HCC.

### Correlation of ALL prognostic signature with clinical features and construction of AS-clinicopathological nomogram

**Fig. 3:**
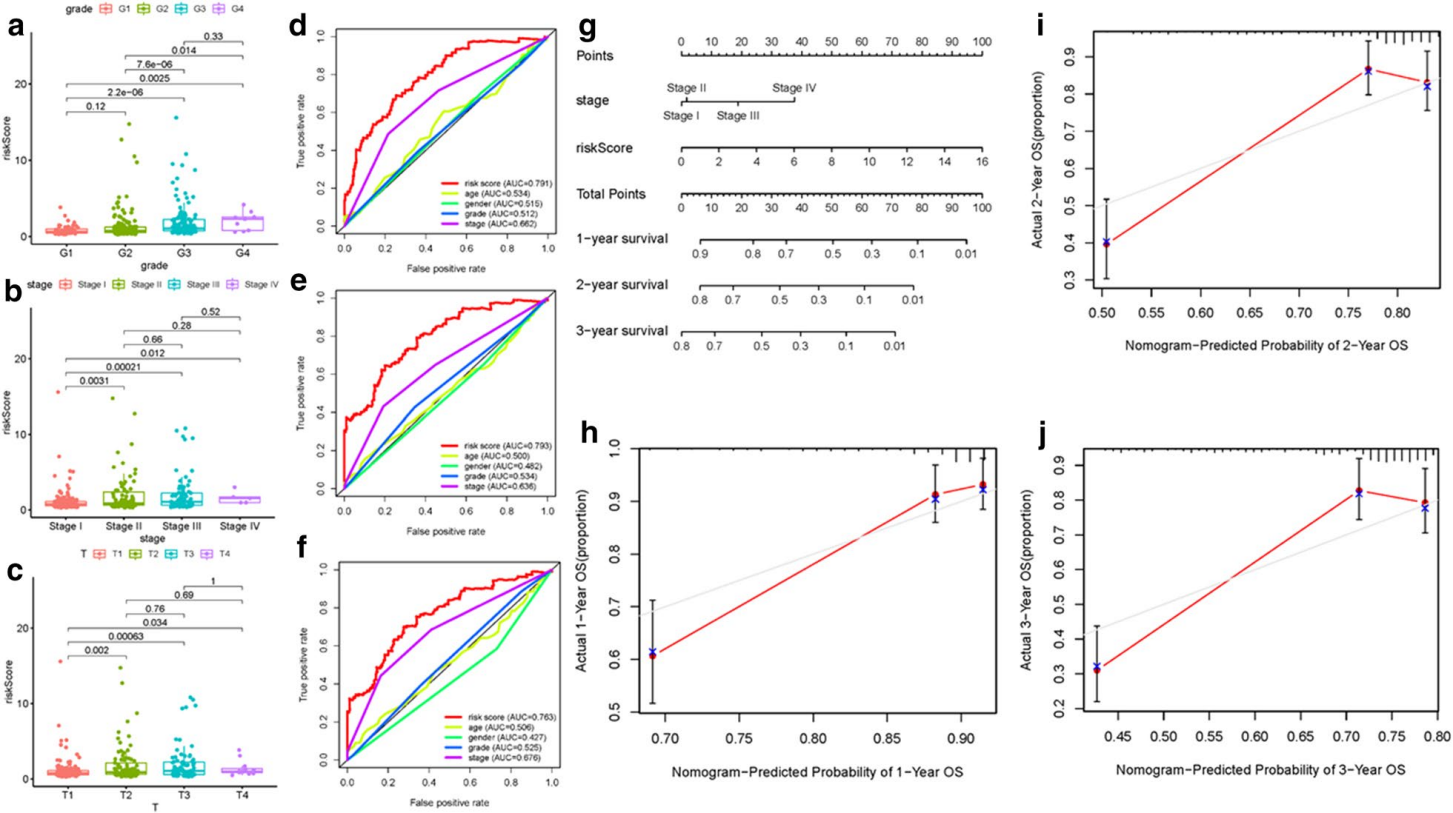
Correlation of ALL prognostic signature with clinical features and construction of AS-clinicopathological nomogram. **a** Correlation of risk score with tumor grade. **b** Correlation of risk score with clinicopathological. **c** Correlation of risk score with T status. **d**–**f** Areas under curves (AUCs) for predicting 1-, 2-, and 3-year survival with different clinical characteristics. **g** Nomogram was assembled by stage and risk signature for predicting survival of HCC patients. **h** One‐year nomogram calibration curves. **i** Two‐year nomogram calibration curves. **j** Three‐year nomogram calibration curves

Differences of risk score among different subtypes according to clinical variables were determined to uncover its clinical significance. The risk score increased significantly with advanced tumor grade (most *p* < 0.05, Fig. 3a), advanced clinicopathological stage (most *p* < 0.05, Fig. 3b) and advanced T category (most *p* < 0.05, Fig. 3c), suggesting risk score was positively correlated with tumor progression. To explore whether ALL prognostic signature was the best prognostic indicator among various clinical characteristics, age, gender, clinicopathological stage, and tumor grade were extracted as the candidate prognosis predictive factors. These clinical variables were consolidated to conduct the AUC curve analysis for 1-, 2-, and 3-year OS and risk signature obtained the most AUC value (Fig. 3d–f). Then, prognostic nomogram including risk score and clinicopathological stage were established to forecast prognosis of patients with HCC (Fig. 3g). Age, gender and tumor grade were rejected out of the nomogram because of their AUCs were less than 0.6. Calibrate curves were approximately diagonal, indicating powerful prognostic predictive ability of 1-, 2- and 3-year OS in our nomogram plot (Fig. 3h–j).

### Correlation of risk score with tumor immune environment characterization

**Fig. 4:**
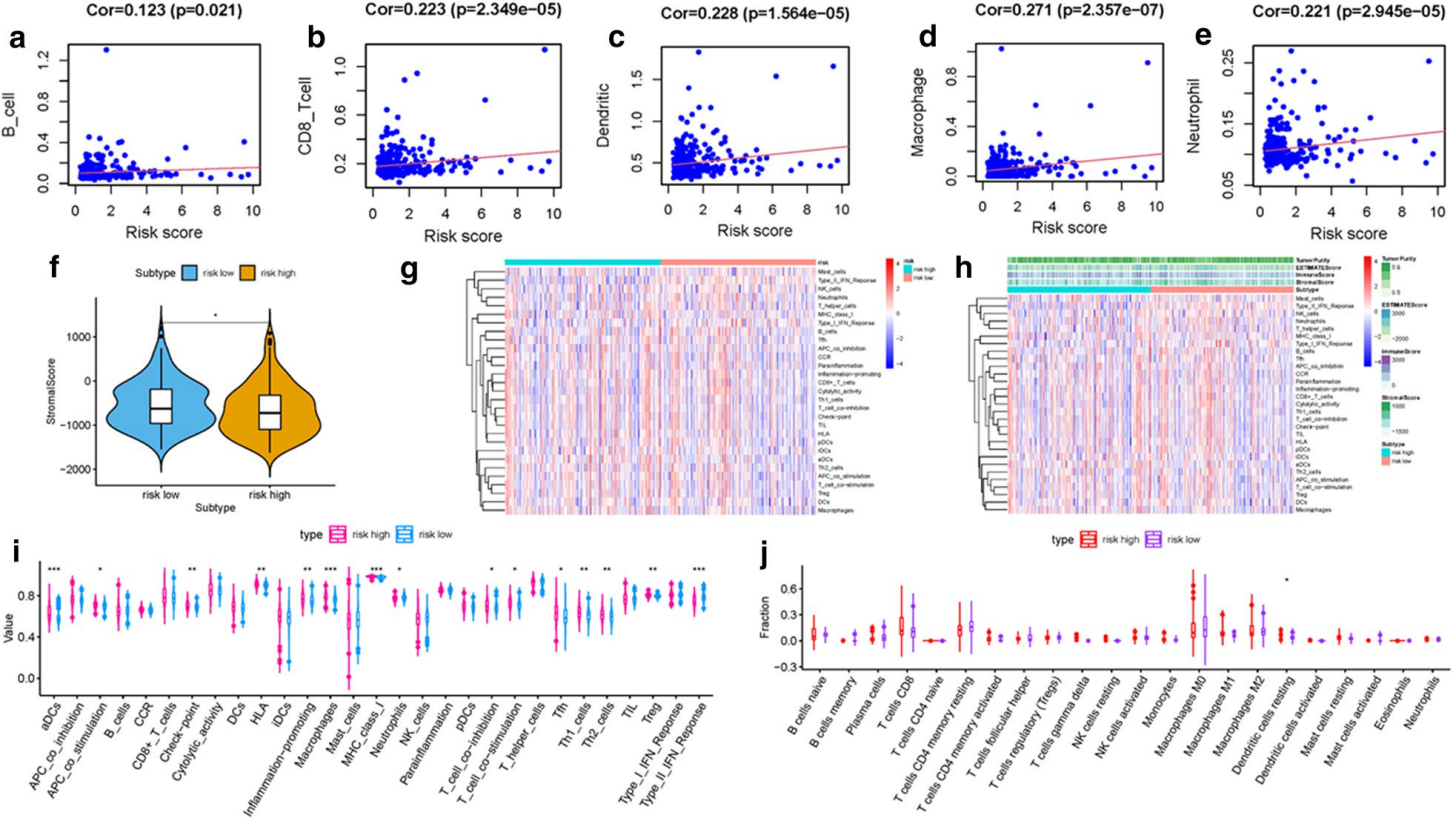
Correlation between infiltrating immune cells and ALL AS-based prognostic signature. **a **Relationship between this signature and B cells. **b** Relationship between this signature and CD8 + T cells. **c** Relationship between this signature and Dendritic cells. **d** Relationship between this signature and Macrophages. **e** Relationship between this signature and Neutrophils. (F) Comparison of stromal score between low- and high-risk groups. **g** Heatmap displayed enrichment of 29 immune signatures of low-/high-risk groups. Blue represents low activity and red represent high activity. **h** Heatmap of 29 immune signatures and immune scores of two different risk score groups. Blue represents low activity and red represent high activity. **i** Distinction of enrichment of immune-related signatures between risk-low and risk-high groups. **j** Difference of infiltrating immune cell subpopulations and levels between low-/high-risk groups

To further examine whether risk score can act as immune indicators, the correlation analysis of prognostic risk score with TICs from TIMER, immune score (calculated using the ESTIMATE algorithm), ssGSEA signatures and TICs subtype and level (calculated via CIBERSORT method) were carried out. Firstly, TIMER results showed that the as-constructed signature exhibited the marked positive association with B cells infiltration (*r* = 0.116; *p* = 0.026), CD8 + T cells infiltration (*r* = 0.223; *p* = 2.349e − 05), Dendritic cells infiltration (r = 0.228; *p* = 1.564e − 05), Macrophages infiltration (*r* = 0.271; *p* = 2.357e − 07) and Neutrophils infiltration (*r* = 0.221; *p* = 2.945e − 05; Fig. 4a–e), indicating high-risk samples were infiltrated more immune cells. Likewise, low-risk patients obtained a higher stromal score which represented less immune infiltration (Fig. 4f). However, there was no significant difference regarding to immune score, estimate score and tumor purity (Additonal file 1: Figures S13A, S13B and S13C). Subsequently, distinction of the immune-related signatures between these two subgroups was presented. Figure 4g and h showed that immune-related signature of each patient with corresponding immune scores in low-/high-risk groups. The results showed that the infiltration of aDCs, Macrophages, Neutrophils, Tfh, Th1 cells, Th2 cells, Tregs, HLA molecule expression level and MCH class I expression, such immune signatures as APC costimulation, T cells costimulation, check-point, inflammation-promoting, and IFN response were significantly increased with increased risk score (Fig. 4i). The CIBERSORT algorithm results indicated that proportion of reseing Dendritic cells was negatively associated with risk score (Fig. 4j). Above results indicated that ALL prognostic signature may provide a novel approach to elucidate the characteristics of immunity regulatory network in HCC.

### Correlation of ALL signature with ICB key molecules

**Fig. 5:**
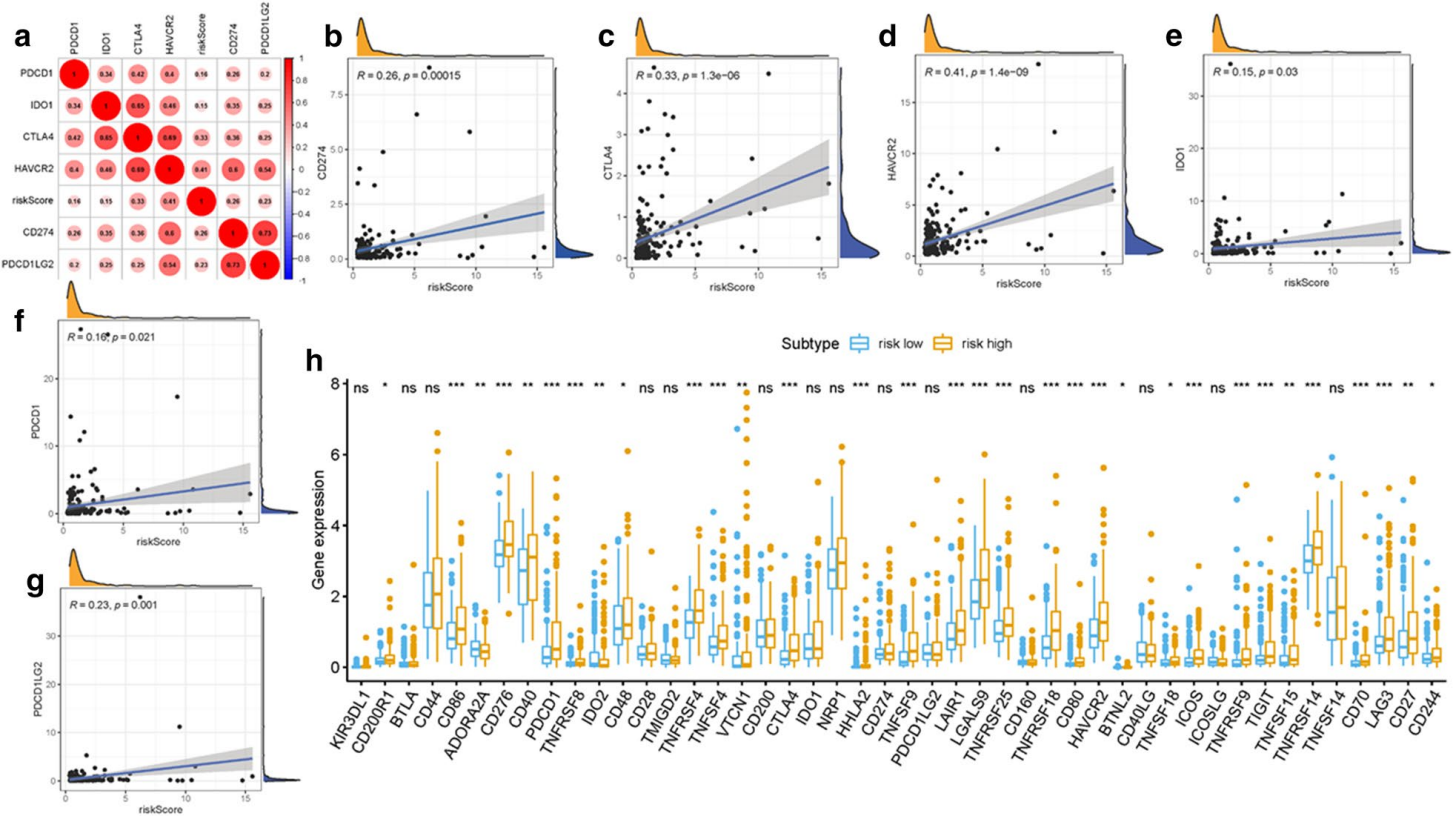
Association between ALL AS-based prognostic signature and crucial immune checkpoint genes. **a** association analyses between immune checkpoint inhibitors CD274, PDCD1, PDCD1LG2, CTLA4, HAVCR2, and IDO1 and risk score. **b** association between risk score and CD274. **c** association between risk score and CTLA4. **d** association between risk score and HAVCR2. **e** association between risk score and IDO1. **f** association between risk score and PDCD1. **g** association between risk score and PDCD1LG2. **h** Comparison of immune checkpoint blockade-related genes expression levels between low-risk group and high-risk groups

With the emergence of immune checkpoint blockade (ICB) therapy, immune checkpoint inhibitors have considerably transformed clinical decision-making in cancer oncology [38–40]. Subsequently, six key immune checkpoint inhibitors genes (PDCD1, CD274, PDCD1LG2, CTLA‐4, HAVCR2, and IDO1) [34–36] were correlated. And the correlation between ICB key targets and ALL prognostic signature was analyzed to reveal the potential player of risk signature in the ICB treatment of HCC (Fig. 5a). The results indicated that ALL prognostic signature was significantly positive correlated to CD274 (*r* = 0.26; *p* = 0.00015), CTLA4 (*r* = 0.33; *p* = 1.3e − 06), HAVCR2 (*r* = 0.41; *p* = 1.4e − 09), IDO1 (*r* = 0.15; *p* = 0.03), PDCD1 (*r* = 0.16; *p* = 0.021) and PDCD1LG2 (*r* = 0.23; *p* = 0.001; Fig. 5b–g). Further correlation analysis presented that 33 of 47 (i.e., PDCD1, CTLA4, etc.,) immune check blockade-associated genes expression levels were significantly upregulated in patients with high-risk (Fig. 5h), suggesting ALL prognostic signature might act as nonnegligible and unfavorable factor in immunotherapy operating.

### ZDHHC16 independently affected prognosis and correlated with ICB key genes

**Fig. 6:**
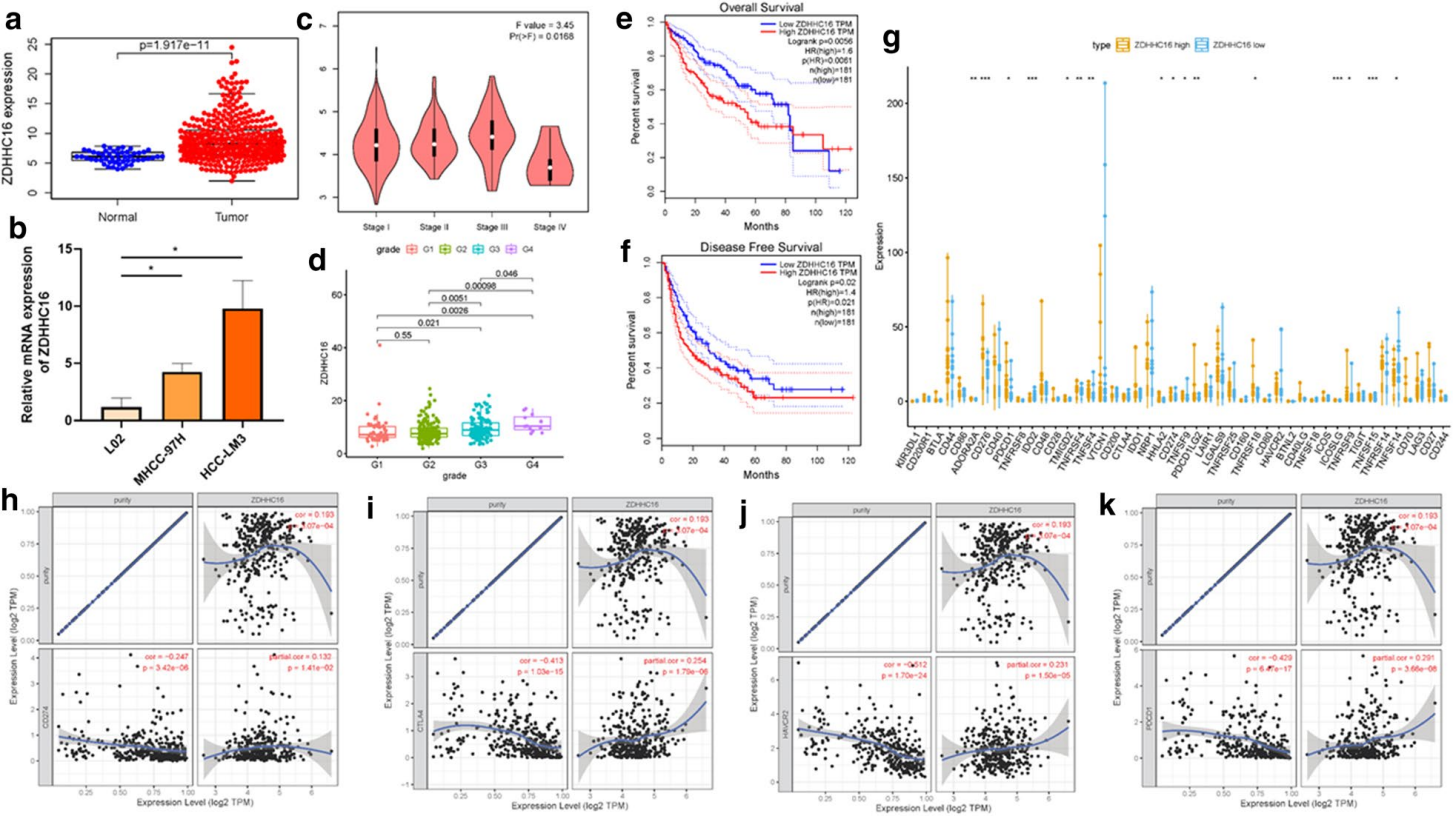
The clinical significance of ZDHHC16 in HCC and in vitro study. ZDHHC16 are overexpressed in HCC tumor tissue (**a**) and HCC cell lines (**b**). **c** The expression of ZDHHC16 had significant difference between major pathological stages. **d** Correlation of risk score with tumor grade. Lower ZDHHC16 level predicts longer overall survival (**e**) and disease-free survival (**f**). **g** Comparison of immune checkpoint blockade-related genes expression levels between low-ZDHHC16 group and high-ZDHHC16 group. **h** Correlation of risk score with CD274. **i** Correlation of risk score with CTLA4. **j** Correlation of risk score with HAVCR2. (K) Correlation of risk score with PDCD1

ZDHHC16 was only one gene whose expression level was upregulated among the prognostic AS-related genes. Therefore, the role of ZDHHC16 in HCC was explored in further experimental validation. ZDHHC16 expression level between normal tissues and tumor samples was compared based on TCGA data. Relative to tumor tissues, ZDHHC16 expression level was lower in adjacent normal specimens (Fig. 6a). Taking advantage of qRT-PCR, ZDHHC16 expression level in two distinct HCC cell lines and human hepatic cell line were determined. Consistent of results of online database, ZDHHC16 was upregulated in cancer cells relative to normal cell (Fig. 6b). The expression level analysis among major pathological stages presented that ZDHHC16 expressed statistical significantly among different pathological stages (Fig. 6c, F = 3.45 and P = 0.0168). Additionally, the later tumor grade, the higher risk score (Fig. 6d, almost *p* < 0.05). To further assess the prognostic value of ZDHHC16 in HCC, Kaplan–Meier analysis were conducted between ZDHHC16 low- and high-expressed patients. As presented in Fig. 6e and f, lower ZDHHC16 expression level significantly suggested longer overall survival time (*p* = 0.0056) and longer disease-free survival time (*p* = 0.02). Besides, 16 of 47 immune check blockade-associated genes (i.e., PDCD1, CTLA4, etc.,) expression levels between low-ZDHHC16 and high-ZDHHC16 groups were significantly dysregulated in between different subgroups (Fig. 6g). Then the correlation between the ZDHHC16 and ICB key targets adjusted by tumor purity using TIMER was analyzed to investigate the potential player of ZDHHC16 in ICB treatment of HCC. TIMER results presented ZDHHC16 was significantly positive correlated to CD274 (*r* = 0.132; *p* = 1.41e − 02), CTLA4 (*r* = 0.254; *p* = 1.79e − 06), HAVCR2 (*r* = 0.231; *p* = 1.50e − 05) and PDCD1 (*r* = 0.291; *p* = 3.66e − 08; Fig. 6h–k), suggesting ZDHHC16 may exert a vital player in ICB treatment of HCC.

### Role of ZDHHC16 in context of TIME

**Fig. 7:**
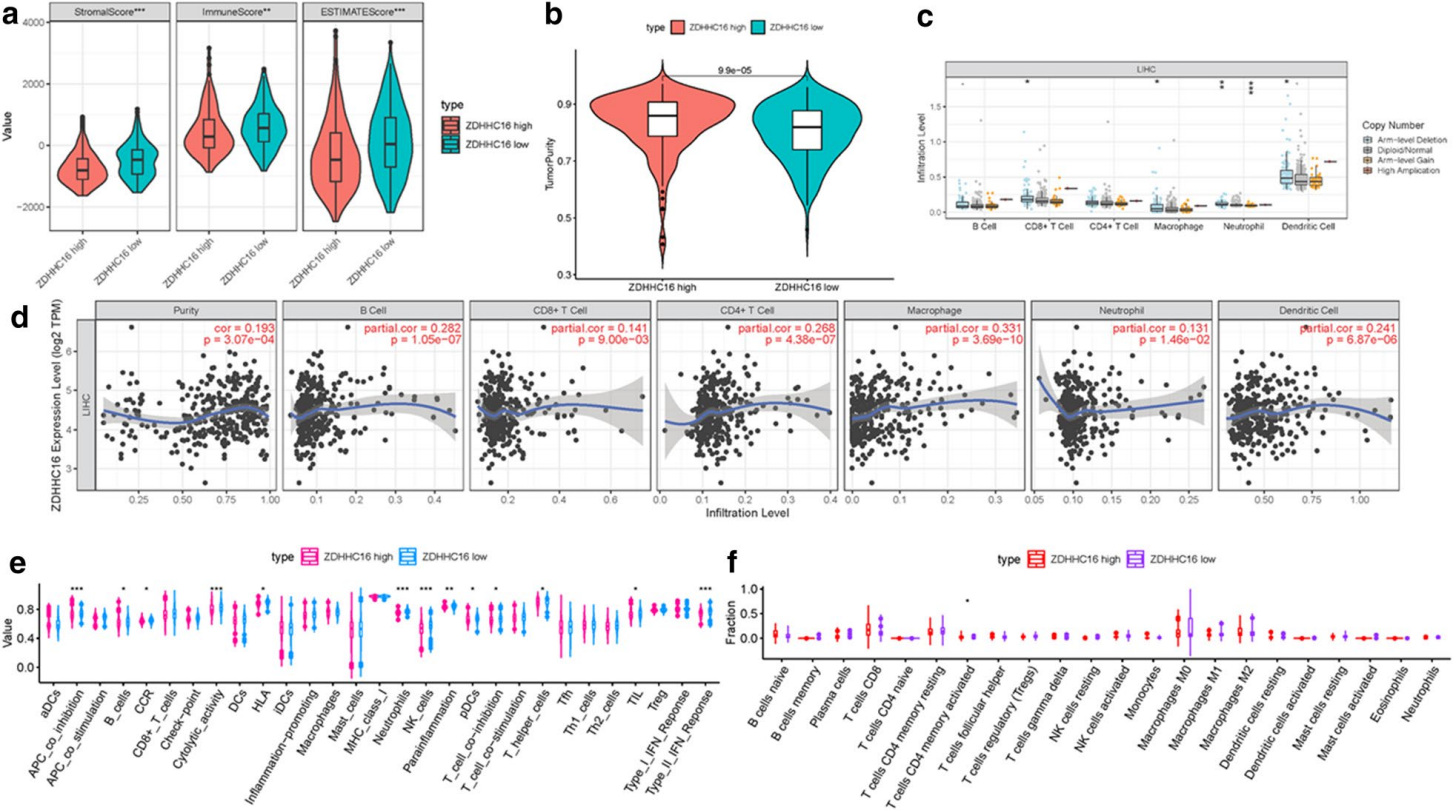
The role of ZDHHC16 in TIME features. **a** Comparison of stromal score, immune score and ESTIMATE score between low-/high-ZDHHC16 groups. **b** Comparison of tumor purity between low-/high-ZDHHC16 groups. **c** Copy number of immune cells in HCC. **d** Relationship between risk score with B cells, CD8 T cells, CD4 T cells, Macrophages, Neutrophils and Dendritic cells. **e** Comparison of ssGSEA enrichment between low-/high-ZDHHC16 groups. **f** Comparison of CIBERSORT results between low-/high-ZDHHC16 groups

To further elucidate the relationship between ZDHHC16 and TIME characteristics in HCC, comprehensive analysis were performed as descripted previously. HCC patients were classified into high-/low- ZDHHC16 subtypes based on the median ZDHHC16 expression level. ESTIMATE results indicated that patients with lower ZDHHC16 expression had a significant higher stromal score and higher immune score relative to patients in high- ZDHHC16 group, suggesting less stromal cells and immune cells in low-risk samples. (Figs. 7a, b). Additionally, arm-level deletion was predominant type of mutation (Fig. 7c). Subsequently, expression level of ZDHHC16 was positively correlated with infiltration of main immune cells types (Fig. 7d). outcomes of ssGSEA showed that the infiltration fraction of B cells, neutrophils, NK cells, T helper cells and TIL, APC co-inhibition, T cell co-inhibition, CCR, cytolytic activity, IFN-response type-I and HLA expression were significantly increased when risk score declining (Fig. 7e). The CIBERSORT analysis results of TCGA cohort showed that the proportion of activated memory CD4 T cells was significantly higher in patients with low-risk (Fig. 7f).

### Development of the SF-AS regulatory network

**Fig. 8:**
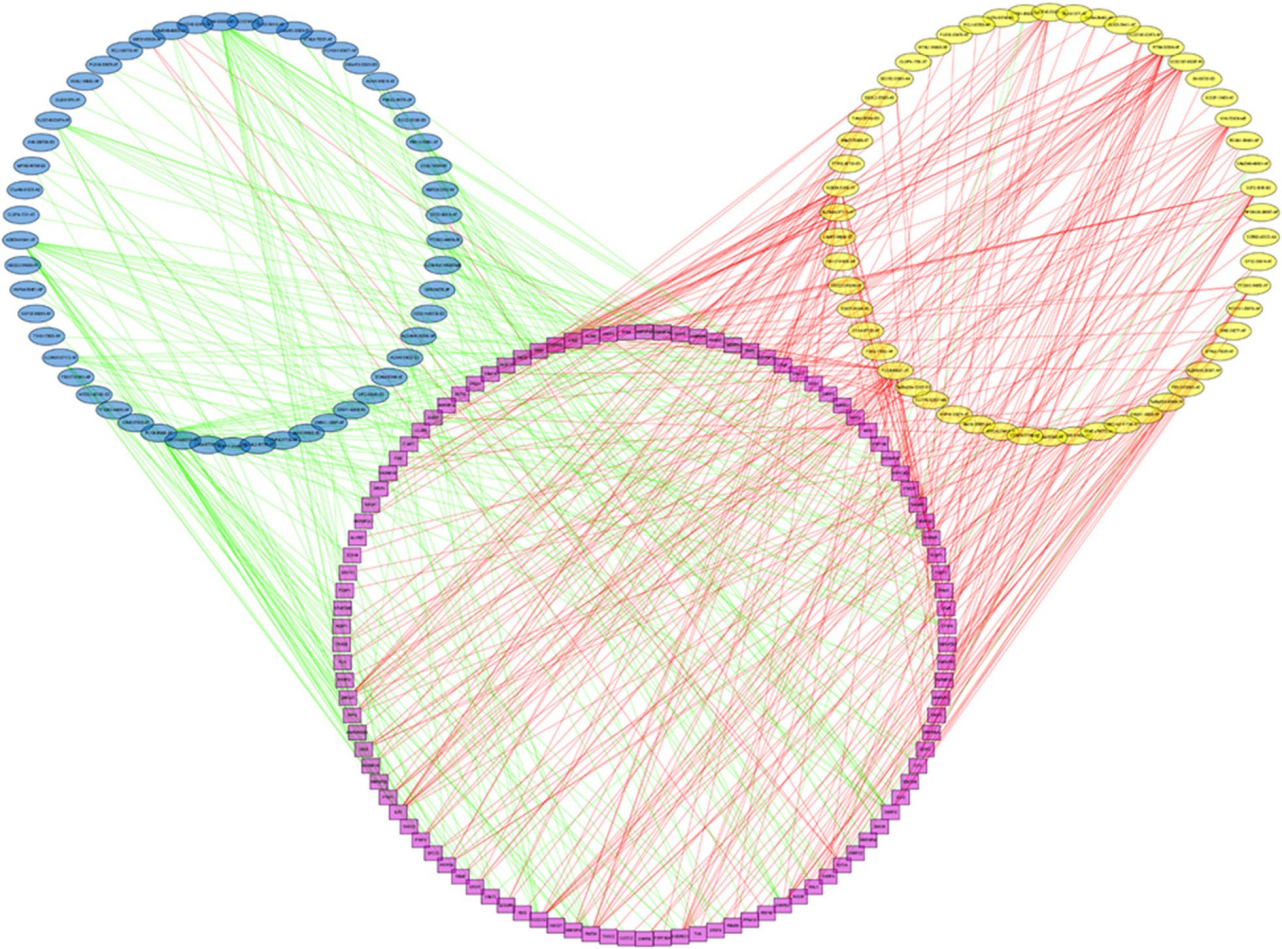
The regulatory network between SFs and survival related AS events. The yellow or blue ellipses indicated the AS events positively or negatively correlated with survival. Purple rectangles represented SFs. The positive/negative correlations (*r* > 0.6 or *r* < − 0.6) between SFs and AS events were indicated with red/green lines

To elucidate the underlying mechanism of AS regulation, a correlation network between the expression level of SFs and the PSI values of prognosis-related AS events was constructed. 55 up-regulated AS events (yellow ellipses), 56 down-regulated AS events (blue ellipses) and 106 SFs (Fig. 8) were identified. In our regulatory network, the top four most significant nodes were termed as the hub SFs or AS events (Additonal file 2: Table S4), including one downregulated AS event (ACAA1-64,022-ES), one upregulated AS events (SCP2-3045-ES) and two SFs (ISY1 and CLK2). As such, these SFs exhibited promising potential to serve as pivotal regulators involving in the dysregulation of AS in HCC, further mediated tumor initiation and progression.

## Discussion

As one of the most common type of malignant tumor, hepatocellular carcinoma (HCC) had high cancer-relevant mortality globally [1–3]. Since such intricate molecular mechanism as genomic complexities and epigenetic diversities, HCC was highly heterogeneous from both clinical standpoint and molecular level [41–43]. A sober reality is that a majority of HCC patients cannot obtain benefit from immunotherapy, due to tumor-promoting condition mediated by immunosuppressive cells (i.e., regulatory T cells, etc.,) [44]. Thus, there is an urgent call to develop powerful prognostic tools for immunotherapeutic outcome prediction, which could contribute novel insight into individual tailored treatment in HCC.

Increasing studies have provided strong evidence to support that AS, which refers to post‑transcriptional modification procedure, function in physiological and pathological process [17]. Notably, abnormally regulated AS events participated well in tumor initiation and development, including HCC [21, 45]. Furthermore, dysregulated expressed genes can be employed as novel prognostic indicator and promising therapeutic targets. However, little to know the correlation of AS events with context of TIME and immunotherapeutic results in HCC.

In current study, AS data was obtained from TCGA SpliceSeq and comprehensive analysis of AS events in HCC samples. Taking advantage of univariate Cox regression analysis, 3294 AS events significantly associated with the survival were identified to explore the prognostic value of AS events. Next, prognostic signatures for HCC patients were proposed using systematic bioinformatic analysis. All eight (AA, AD, AP, AT, ES, ME, RI, and ALL) prognostic predictive signatures constructed by AS patterns presented powerful capability for the prognostic prediction in HCC. Notably, these AS-based prognostic signatures were robustly demonstrated by K-M survival analysis, ROC curve and Cox regression analysis. Furthermore, this signature retained excellent prognostic performance when HCC cases divided into groups based on clinicopathological factors. To transform ALL risk model into further clinical practice, a nomogram graph including prognostic signature with clinicopathological stage was plotted, and there was high consistence between predicted outcome and actual outcome. Besides, the most significant associated SF-AS regulatory network in the TCGA LIHC cohort were screened.

To reveal the role of AS events in the context of TIME in HCC, TIMER database, ESTIMATE algorithm, ssGSEA method and CIBERSORT analysis were conducted. These results presented that high-risk score group was marked with high infiltration of immune cells and more activated immune condition, which might promote immune recognition and trigger anti-tumor effect. These outcomes implied that risk score might facilitate immunotherapy results prediction. Since no ICB treatment dataset in HCC cohort, it was unable to investigate the relationship between risk score and ICB immunotherapy response. Then, risk score was positively and significantly correlated with six ICB key targets (i.e., CD274 and CTLA4) and 33 (i.e., PDCD1LG2, etc.,) immune check blockade-associated genes expression levels, which imply that risk score might contribute to strategizing the tailored immunotherapy.

ZDHHC16 was a DHHC encoding protein, which was tightly correlated with protein palmitoylation in previous researches [46]. Wei Shi et al. reported that ZDHHC16 acted as a vital regulator in the process of NSPCs proliferation [47]. Li Jian et al. uncovered pivotal players for ZDHHC16 in the regulation of DNA damage responses and Atm activation(48). To date, little to know about the role of ZDHHC16 in tumors, especially in HCC. This study presents that ZDHHC16 is significantly upregulated in HCC cell lines and suggested poor prognosis in HCC. ZDHHC16 expression was significantly positive associated with clinicopathological stage, tumor grade and ICB immunotherapy key genes (i.e., CD274, CTLA4, HAVCR2 and PDCD1, etc.). Nevertheless, further research needed to explore the underlying biological roles of ZDHHC16.

Collectively, subjects with higher risk score or higher ZDHHC16 expression level presented more abundance of immune cells in tumor environment, suggesting activated immune phenotype, but shorter overall survival time. It could be speculated that anti-tumor effect of immune cells may be affected by immune checkpoint blockade pathways given risk score was correlated with immune checkpoint blockade targets expression.

Compared this research with existed studies that explored the novel prognostic factor in HCC, some superiorities of our research should be noted. Firstly, this study contributed to investigate the potential roles of AS events in formation of TIME diversity and complexity and ICB treatment prediction, which has not been elucidated before this study. Additionally, ESTIMATE R package, ssGSEA algorithm and CIBERSORT method and TIMER database exploration were performed to uncover the comprehensive landscape of TIME in HCC. Finally, to our knowledge, this work is the first placed emphasis on the biological functions of ZDHHC16 in HCC.

## Conclusion

Collectively, systematical analyses in prognosis predictive value of RNA splicing patterns were performed, which was designed to strengthen prognosis prediction in HCC. It is worthwhile mentioned that novel and robust prognostic nomogram to predict outcome quantitatively was established, which exhibited encouraging potential into clinical application. Besides, the AS-SFs regulatory network suggested promising targets of the anti-tumor therapy in HCC. The comprehensive bioinformatic analysis of AS events robustly linked the AS atlas with TIME characterization and immunotherapy in HCC. Nevertheless, these findings should be validated in further experimental and clinical exploration which focusing on HCC tumorigenesis and progression mechanisms and the impacts of these AS events.

## Supplementary Information

**Additional file 1**: **Figure S1**. Overall research design. Flow-process diagram presenting the process of comprehensive analysis. **Figure S2**. (A)The upset plot of gene interactions among the seven types of AS events in TCGA LIHC cohort. (B) The upset plot of gene interactions among the seven types of survival relevant AS events. **Figure S3.** LASSO coefficient of survival relevant AS events. (A) AA. (B) AD. (C) AP. (D) AT. (E) ES. (F) ME. (G) RI. **Figure S4**. A graph of the error rate of cross-validation. (A) AA. (B) AD. (C) AP. (D) AT. (E) ES. (F) ME. (G) RI. **Figure S5**. (A) Heatmap of the AA events PSI value in HCC. The color from red to blue shows a trend from high expression to low expression. (B) Distribution of AA prognostic signature risk score. (C) The survival status and duration of HCC patients in AA prognostic signature. (D) Heatmap of the AD events PSI value in HCC. The color from red to blue shows a trend from high expression to low expression. (E) Distribution of AD prognostic signature risk score. (F) The survival status and duration of HCC patients in AD prognostic signature. **Figure S6**. (A) Heatmap of the AP events PSI value in HCC. The color from red to blue shows a trend from high expression to low expression. (B) Distribution of AP prognostic signature risk score. (C) The survival status and duration of HCC patients in AP prognostic signature. (D) Heatmap of the AT events PSI value in HCC. The color from red to blue shows a trend from high expression to low expression. (E) Distribution of AT prognostic signature risk score. (F) The survival status and duration of HCC patients in AT prognostic signature. **Figure S7**. (A) Heatmap of the ES events PSI value in HCC. The color from red to blue shows a trend from high expression to low expression. (B) Distribution of ES prognostic signature risk score. (C) The survival status and duration of HCC patients in ES prognostic signature. (D) Heatmap of the ME events PSI value in HCC. The color from red to blue shows a trend from high expression to low expression. (E) Distribution of ME prognostic signature risk score. (F) The survival status and duration of HCC patients in ME prognostic signature. **Figure S8**. (A) Heatmap of the RI events PSI value in HCC. The color from red to blue shows a trend from high expression to low expression. (B) Distribution of RI prognostic signature risk score. (C) The survival status and duration of HCC patients in RI prognostic signature. **Figure S9**. (A) Kaplan–Meier curve presenting survival in AA prognostic signature. (B) ROC analysis of the risk scores in AA prognostic signature. (C) Kaplan–Meier curve presenting survival in AD prognostic signature. (D) ROC analysis of the risk scores in AD prognostic signature. (E) Kaplan–Meier curve presenting survival in AP prognostic signature. (F) ROC analysis of the risk scores in AP prognostic signature. (G) Kaplan–Meier curve presenting survival in AT prognostic signature. (H) ROC analysis of the risk scores in AT prognostic signature. **Figure S10**. (A) Kaplan–Meier curve presenting survival in ES prognostic signature. (B) ROC analysis of the risk scores in ES prognostic signature. (C) Kaplan–Meier curve presenting survival in ME prognostic signature. (D) ROC analysis of the risk scores in ME prognostic signature. (E) Kaplan–Meier curve presenting survival in RI prognostic signature. (F) ROC analysis of the risk scores in RI prognostic signature. **Figure S11**. (A) Univariate Cox regression analyses in AA prognostic signature. (B) Univariate Cox regression analyses in AD prognostic signature. (C) Univariate Cox regression analyses in AP prognostic signature. (D) Univariate Cox regression analyses in AT prognostic signature. (E) Multivariate Cox regression analyses in AA prognostic signature. (F) Multivariate Cox regression analyses in AD prognostic signature. (G) Multivariate Cox regression analyses in AP prognostic signature. (H) Multivariate Cox regression analyses in AT prognostic signature. (I) Univariate Cox regression analyses in ES prognostic signature. (J) Univariate Cox regression analyses in ME prognostic signature. (K) Univariate Cox regression analyses in RI prognostic signature. (L) Multivariate Cox regression analyses in ES prognostic signature. (M) Multivariate Cox regression analyses in ME prognostic signature. (N) Multivariate Cox regression analyses in RI prognostic signature. **Figure S12**. Kaplan–Meier survival analysis for multiple HCC subgroups according to the ALL signature stratified by clinical variables. (A-B) Stage. (C-D) T status. (E-F) Gender. (G-H) Tumor grade. (I-J) Age. (K) N status. (L) M status. **Figure S13**. (A) Comparison of the ESTIMATE score between risk score low/high groups. (B) Comparison of the immune score between risk score low/high groups. (C) Comparison of the tumor purity between risk score low/high groups.

**Additional file 2**: **Table S1**. Univariate Cox regression analysis between AS events and survial. **Table S2**. Formula prognostic signature of HCC. **Table S3**. The list of splicing factor (SF) genes. **Table S4**. Results of correlation between AS events and SFs.

## Data Availability

The study was based on the data available at TCGA(https://www.cancer.gov/tcga).

## Abbreviations

AA: Alternate acceptor site
AD: Alternate donor site
AP: Alternate promoter
AT: Alternate terminator
AUC: Area under the curve
AS: Alternative splicing
BCLC: Barcelona Clinic Liver Cancer
CTLA‐4: Cytotoxic T‐lymphocyte antigen 4
CD274: Also known as PD-L1
DMEM: Dulbecco’s minimum essential media
ES: Exon skip
FBS: Fetal bovine serum
GAPDH: Glyceraldehyde-3-phosphate dehydrogenase
GO: Gene ontology
HCC: Hepatocellular carcinoma
HAVCR2: Also known as TIM3
IDO1: Indoleamine 2,3‐dioxygenase 1
ICB: Immune checkpoint blockade
LASSO: Least absolute shrinkage and selection operator
ME: Mutually exclusive exons
OS: Overall survival
PD‐1: Programmed Cell Death 1
PD‐L1: Programmed Cell Death-Ligand 1
PD‐L2: Programmed Cell Death-Ligand 2
PDCD1: Also known as PD-1
PDCD1LG2: Also known as PD‐L2
qRT-PCR: Quantitative real-time polymerase chain reaction
RI: Retained intron
RNA: Ribonucleic Acid
ROC: Receiver operating characteristic
SFs: Splicing factors
ssGSEA: Single-sample gene set enrichment analysis
TCGA: The Cancer Genome Atlas
TICs: Tumor‐infiltrating immune cells
TIME: Tumor immune microenvironment
TIMER: Tumor immune estimation resource
TIM‐3: T‐cell immunoglobulin domain and mucin domain‐containing molecule‐3
TNM: Tumor, node, metastasis
