# Correction to: Immunological significance of prognostic alternative splicing signature in hepatocellular carcinoma

**DOI:** 10.1186/s12935-021-02139-9

**Published:** 2021-08-24

**Authors:** Qianhui Xu, Hao Xu, Rongshan Deng, Nanjun Li, Ruiqi Mu, Zhixuan Qi, Yunuo Shen, Zijie Wang, Jingchao Wen, Jiaxin Zhao, Di Weng, Wen Huang

**Affiliations:** 1 The Second Affiliated Hospital and Yuying Children’s Hospital of Wenzhou Medical University, No 109. Xueyuan West Road, Wenzhou, 325000 Zhejiang China; 2 Zhejiang University School of Medicine, Hangzhou, 310009 Zhejiang China

## Correction to: Cancer Cell Int (2021) 21:190 https://doi.org/10.1186/s12935-021-01894-z

Following the publication of the original article [1], we were notified that Additional file 1 only contained the captions of the additional figures, and not the figures themselves.

The original article has now been corrected.
