# Supplementary material for: Immunological significance of prognostic alternative splicing signature in hepatocellular carcinoma
Source: Cancer Cell Int. 2021 Apr 1;21:190. doi: 10.1186/s12935-021-01894-z (PMC8017877; doi:10.1186/s12935-021-01894-z)
Supplement: Supplementary file 1 — Additional file 1: Figure S1. Overall research design. Flow-process diagram presenting the process of comprehensive analysis. Figure S2. (A)The upset plot of gene interactions among the seven types of AS events in TCGA LIHC cohort. (B) The upset plot of gene interactions among the seven types of survival relevant AS events. Figure S3. LASSO coefficient of survival relevant AS events. (A) AA. (B) AD. (C) AP. (D) AT. (E) ES. (F) ME. (G) RI. Figure S4. A graph of the error rate of cross-validation. (A) AA. (B) AD. (C) AP. (D) AT. (E) ES. (F) ME. (G) RI. Figure S5. (A) Heatmap of the AA events PSI value in HCC. The color from red to blue shows a trend from high expression to low expression. (B) Distribution of AA prognostic signature risk score. (C) The survival status and duration of HCC patients in AA prognostic signature. (D) Heatmap of the AD events PSI value in HCC. The color from red to blue shows a trend from high expression to low expression. (E) Distribution of AD prognostic signature risk score. (F) The survival status and duration of HCC patients in AD prognostic signature. Figure S6. (A) Heatmap of the AP events PSI value in HCC. The color from red to blue shows a trend from high expression to low expression. (B) Distribution of AP prognostic signature risk score. (C) The survival status and duration of HCC patients in AP prognostic signature. (D) Heatmap of the AT events PSI value in HCC. The color from red to blue shows a trend from high expression to low expression. (E) Distribution of AT prognostic signature risk score. (F) The survival status and duration of HCC patients in AT prognostic signature. Figure S7. (A) Heatmap of the ES events PSI value in HCC. The color from red to blue shows a trend from high expression to low expression. (B) Distribution of ES prognostic signature risk score. (C) The survival status and duration of HCC patients in ES prognostic signature. (D) Heatmap of the ME events PSI value in HCC. The color from red to blue shows a tre [file 12935_2021_1894_MOESM1_ESM.pdf]

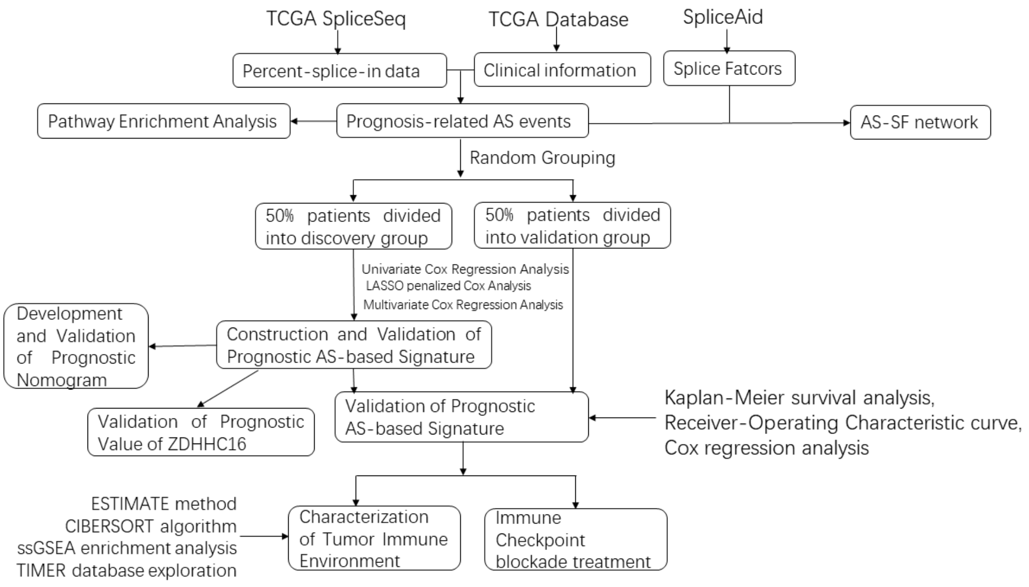

Figure S1. Overall research design. Flow-process diagram presenting the process of comprehensive analysis.

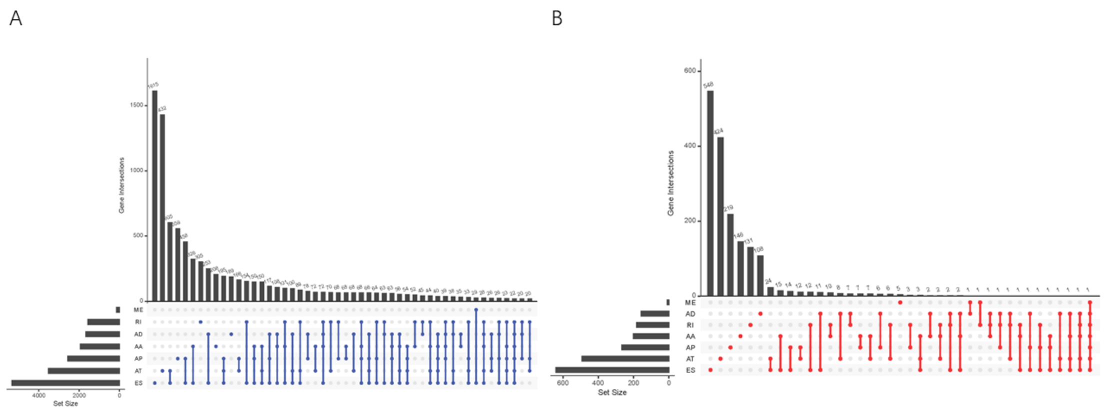

Figure S2: (A)The upset plot of gene interactions among the seven types of AS events in TCGA LIHC cohort. (B) The upset plot of gene interactions among the seven types of survival relevant AS events.

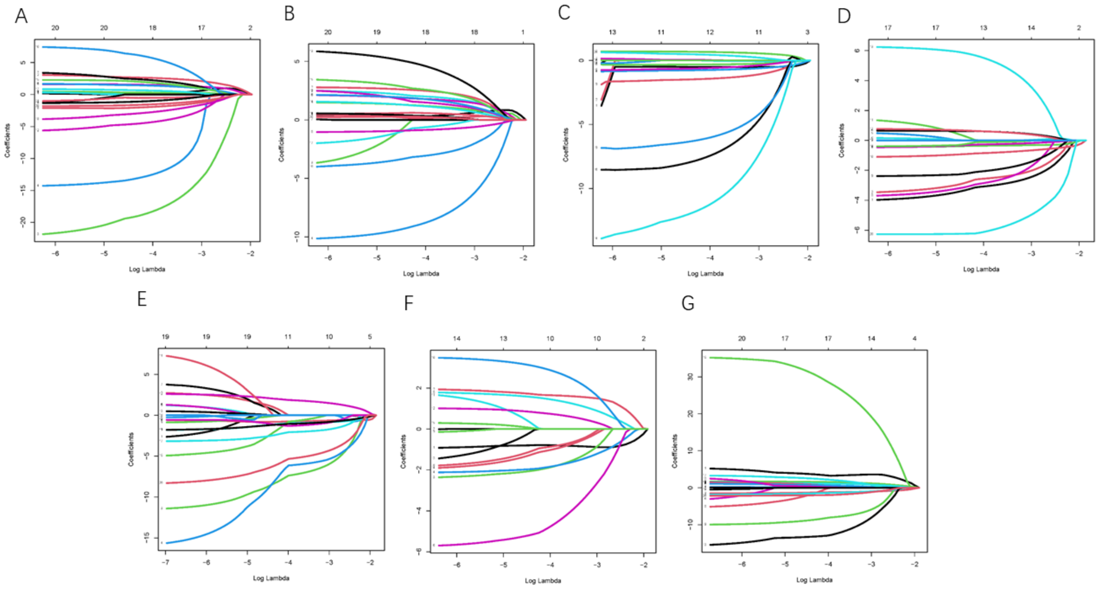

Figure S3: LASSO coefficient of survival relevant AS events. (A) AA. (B) AD. (C) AP. (D) AT. (E) ES. (F) ME. (G) RI.

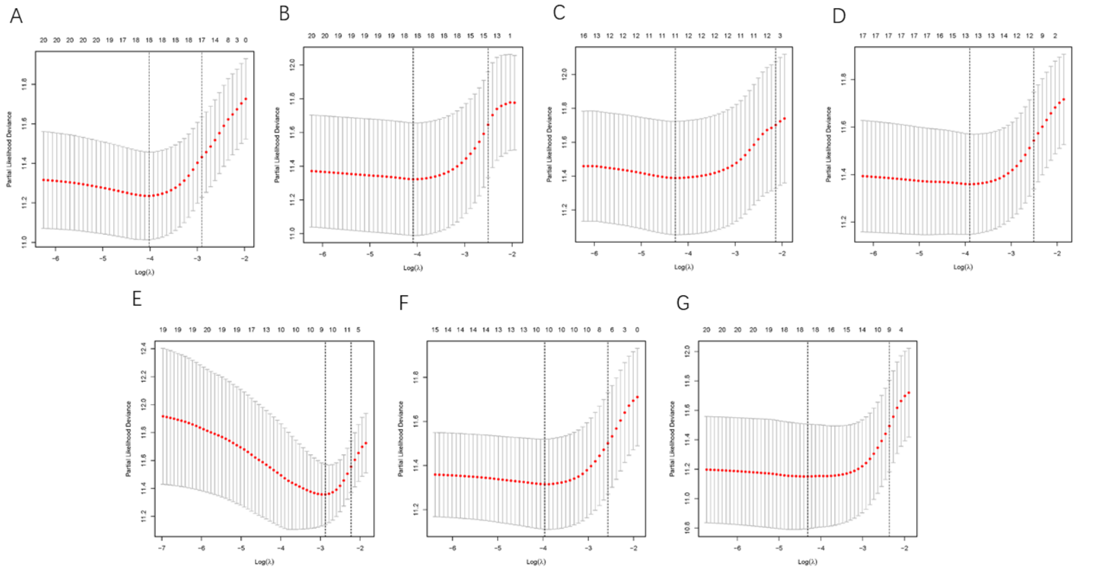

Figure S4: A graph of the error rate of cross-validation. (A) AA. (B) AD. (C) AP. (D) AT. (E) ES. (F) ME. (G) RI.

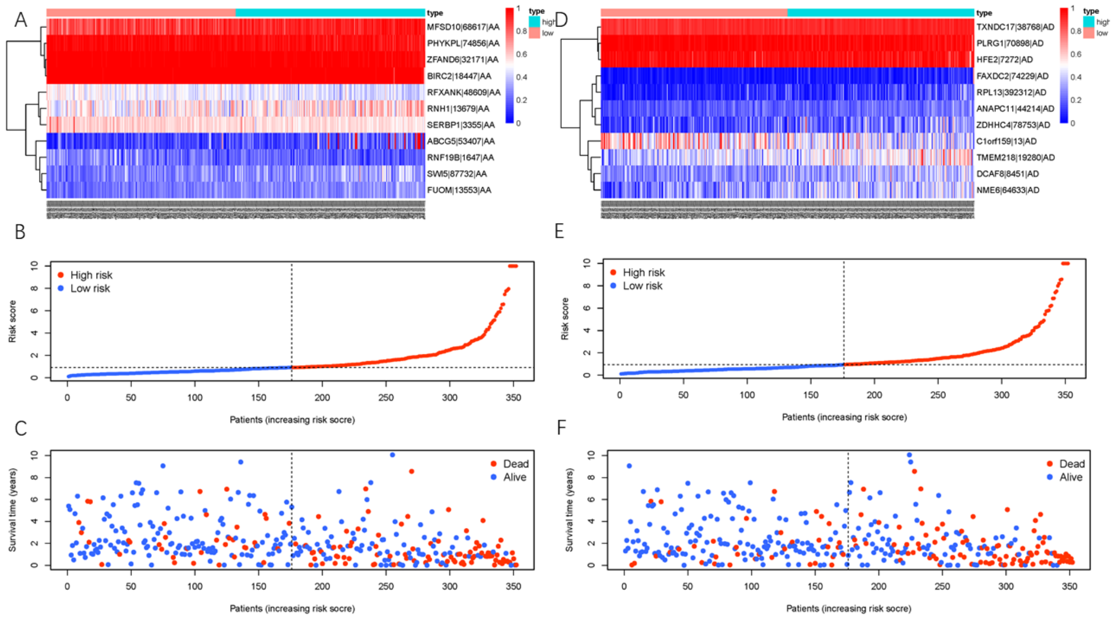

Figure S5: (A) Heatmap of the AA events PSI value in HCC. The color from red to blue shows a trend from high expression to low expression. (B) Distribution of AA prognostic signature risk score. (C) The survival status and duration of HCC patients in AA prognostic signature. (D) Heatmap of the AD events PSI value in HCC. The color from red to blue shows a trend from high expression to low expression. (E) Distribution of AD prognostic signature risk score. (F) The survival status and duration of HCC patients in AD prognostic signature.

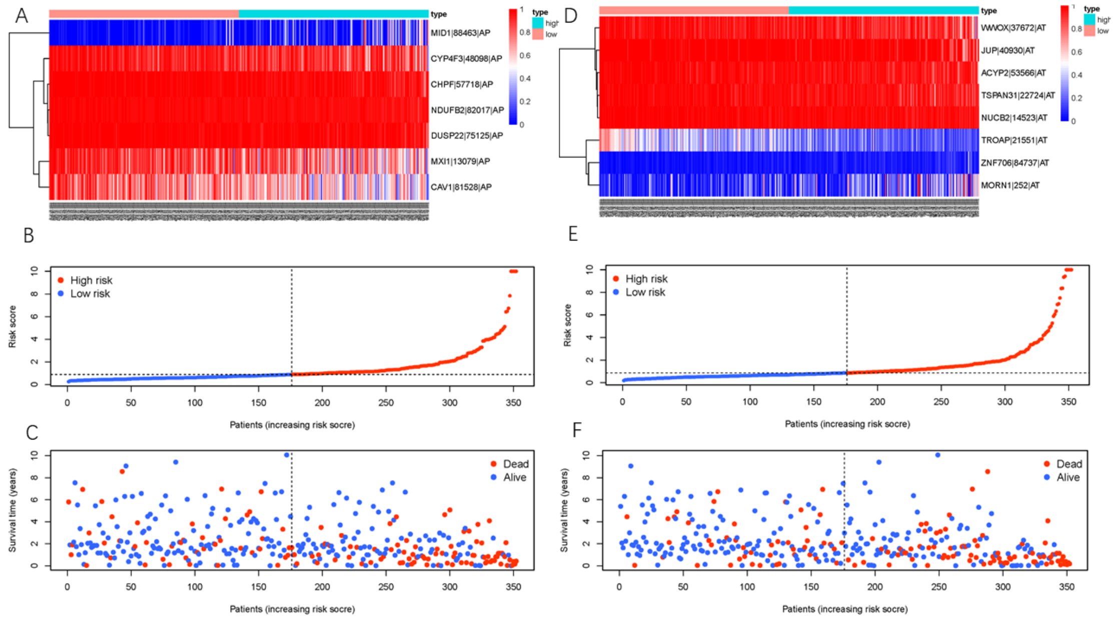

Figure S6: (A) Heatmap of the AP events PSI value in HCC. The color from red to blue shows a trend from high expression to low expression. (B) Distribution of AP prognostic signature risk score. (C) The survival status and duration of HCC patients in AP prognostic signature. (D) Heatmap of the AT events PSI value in HCC. The color from red to blue shows a trend from high expression to low expression. (E)

Distribution of AT prognostic signature risk score. (F) The survival status and duration of HCC patients in AT prognostic signature.

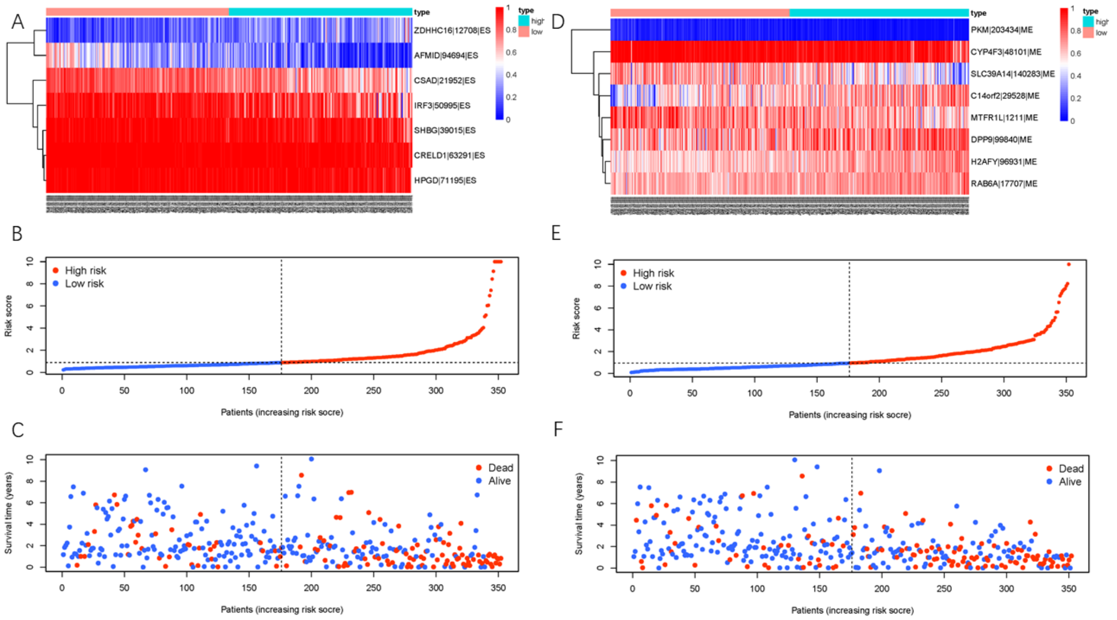

Figure S7: (A) Heatmap of the ES events PSI value in HCC. The color from red to blue shows a trend from high expression to low expression. (B) Distribution of ES prognostic signature risk score. (C) The survival status and duration of HCC patients in ES prognostic signature. (D) Heatmap of the ME events PSI value in HCC. The color from red to blue shows a trend from high expression to low expression. (E) Distribution of ME prognostic signature risk score. (F) The survival status and duration of HCC patients in ME prognostic signature.

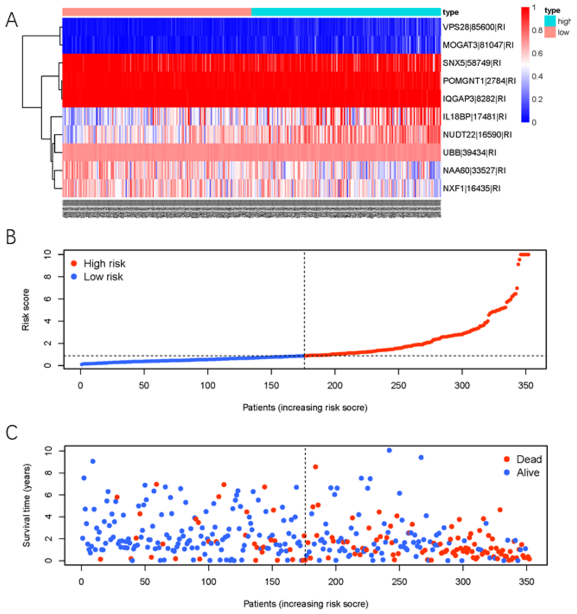

Figure S8: (A) Heatmap of the RI events PSI value in HCC. The color from red to blue shows a trend from high expression to low expression. (B) Distribution of RI prognostic signature risk score. (C) The survival status and duration of HCC patients

in RI prognostic signature.

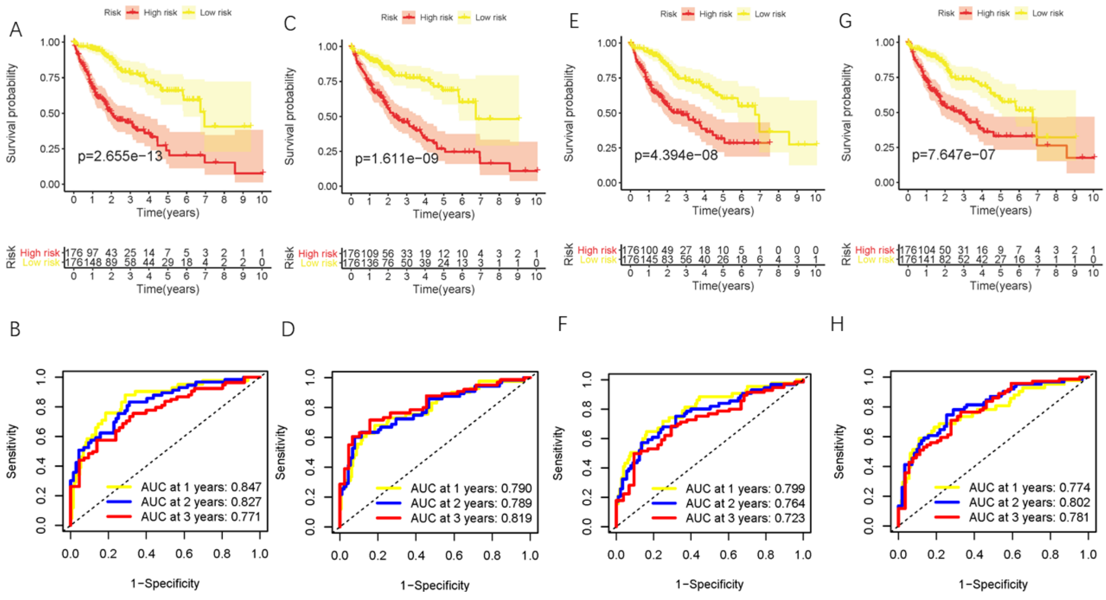

Figure S9: (A) Kaplan–Meier curve presenting survival in AA prognostic signature. (B) ROC analysis of the risk scores in AA prognostic signature. (C) Kaplan–Meier curve presenting survival in AD prognostic signature. (D) ROC analysis of the risk scores in AD prognostic signature. (E) Kaplan–Meier curve presenting survival in AP prognostic signature. (F) ROC analysis of the risk scores in AP prognostic signature. (G) Kaplan–Meier curve presenting survival in AT prognostic signature. (H) ROC analysis of the risk scores in AT prognostic signature.

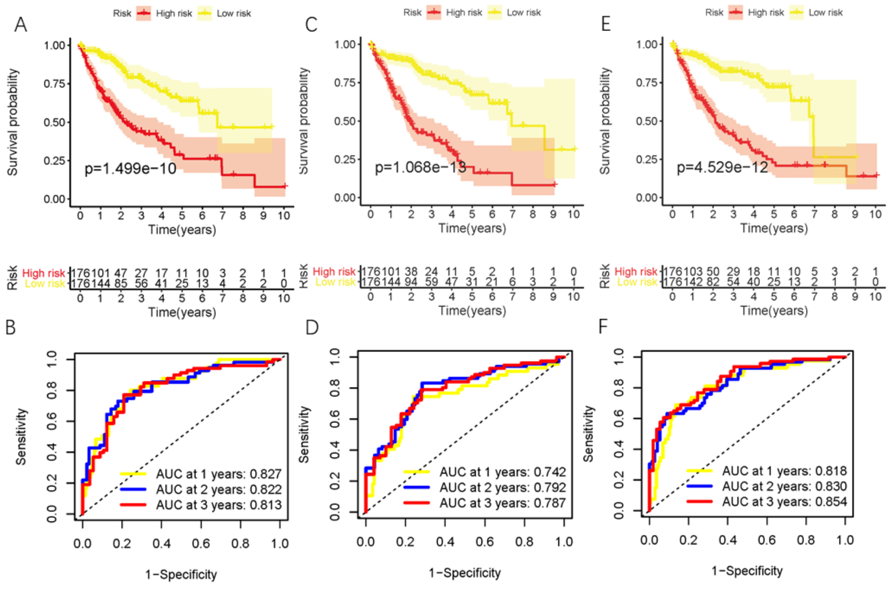

Figure S10: (A) Kaplan–Meier curve presenting survival in ES prognostic signature. (B) ROC analysis of the risk scores in ES prognostic signature. (C) Kaplan–Meier curve presenting survival in ME prognostic signature. (D) ROC analysis of the risk scores in ME prognostic signature. (E) Kaplan–Meier curve presenting survival in RI

prognostic signature. (F) ROC analysis of the risk scores in RI prognostic signature.

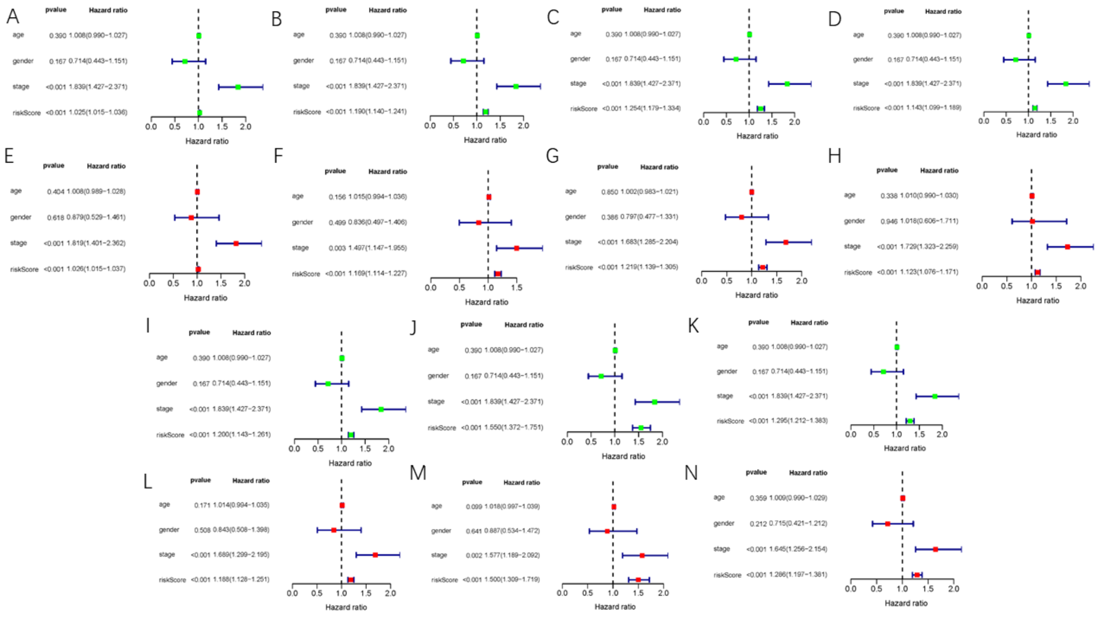

Figure S11: (A) Univariate Cox regression analyses in AA prognostic signature. (B) Univariate Cox regression analyses in AD prognostic signature. (C) Univariate Cox regression analyses in AP prognostic signature. (D) Univariate Cox regression analyses in AT prognostic signature. (E) Multivariate Cox regression analyses in AA prognostic signature. (F) Multivariate Cox regression analyses in AD prognostic signature. (G) Multivariate Cox regression analyses in AP prognostic signature. (H) Multivariate Cox regression analyses in AT prognostic signature. (I) Univariate Cox regression analyses in ES prognostic signature. (J) Univariate Cox regression analyses in ME prognostic signature. (K) Univariate Cox regression analyses in RI prognostic signature. (L) Multivariate Cox regression analyses in ES prognostic signature. (M) Multivariate Cox regression analyses in ME prognostic signature. (N) Multivariate Cox regression analyses in RI prognostic signature.

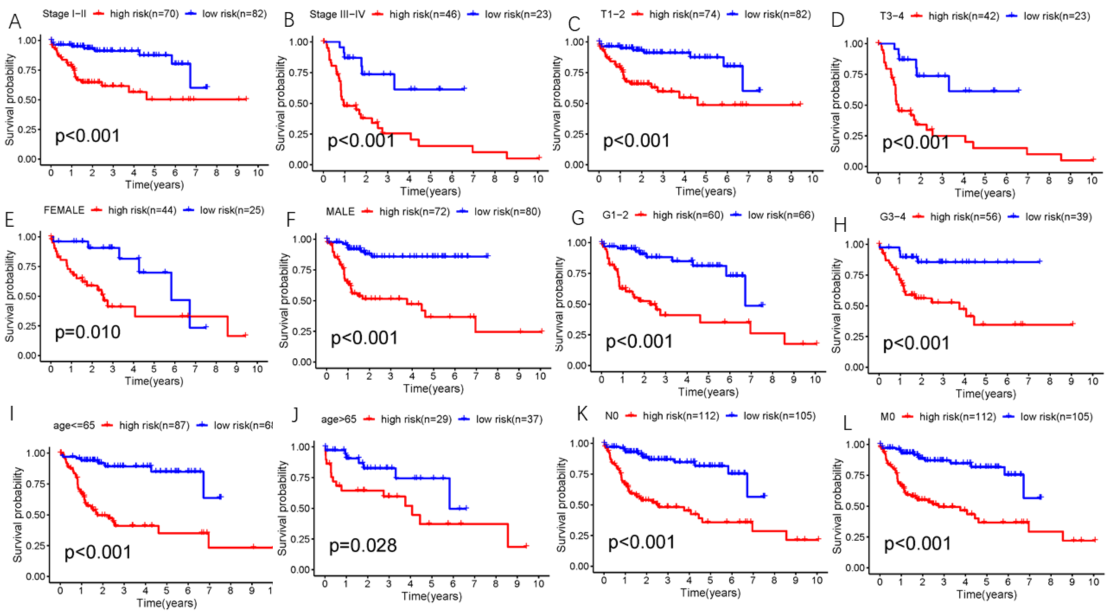

Figure S12: Kaplan–Meier survival analysis for multiple HCC subgroups according to the ALL signature stratified by clinical variables. (A-B) Stage. (C-D) T status. (E-F) Gender. (G-H) Tumor grade. (I-J) Age. (K) N status. (L) M status.

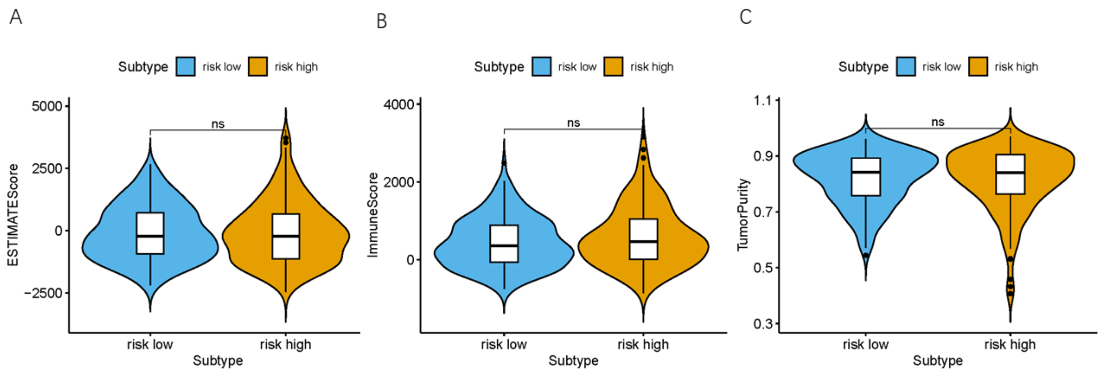

Figure S13: (A) Comparison of the ESTIMATE score between risk score low/high groups. (B) Comparison of the immune score between risk score low/high groups. (C) Comparison of the tumor purity between risk score low/high groups.
